# Protein regulatory network mediated by palmitoylation modifications in the pathological progression of Parkinson’s disease: a narrative review

**DOI:** 10.3389/fimmu.2025.1615001

**Published:** 2025-07-09

**Authors:** Jingjing Liu, Shanshan Wang, Lei Fan, Xin Zhou, Sen Zhang, Qinglu Wang, Panpan Dong, Bo Yu

**Affiliations:** ^1^ Graduate School of Education, Shandong Sport University, Jinan, China; ^2^ Department of Neurology, Zibo 148 Hospital, Zibo, Shandong, China; ^3^ Department of Rehabilitation, Liuzhou People’s Hospital, Liuzhou, China; ^4^ College of Basic Medicine, Qilu Medical University, Zibo, Shandong, China

**Keywords:** Parkinson’s disease, palmitoylation, α-synuclein, synaptotagmin-11, NLRP3 inflammasome

## Abstract

Palmitoylation is a reversible lipid modification regulated by palmitoyl transferases and acyl-protein thioesterases, in which palmitic acid is attached to protein cysteine residues. This modification plays a pivotal role in modulating membrane localization and protein stability, and its dysregulation is closely associated with various neurodegenerative diseases, including Parkinson’s disease (PD). In PD, synaptotagmin-11, encoded by the PD risk gene *SYT11*, has been shown to reduce physiological α-synuclein (α-syn) tetramer formation while promoting the aggregation-prone monomeric form in a palmitoylation-dependent manner. In the context of PD, inflammation generally precedes the abnormal aggregation of α-syn and the degeneration of dopaminergic neurons (DA). Microglial activation, regarded as an inflammatory state, is facilitated by the palmitoylation-dependent localization of NLRP3 to the trans-Golgi network, which promotes the activation and expression of the NLRP3 inflammasome, leading to DA neuron loss. Additionally, the DJ-1 protein, encoded by the risk gene *PARK7*, and the dopamine transporter both undergo palmitoylation and may contribute to disease progression. This review summarizes the emerging link between protein palmitoylation and PD pathogenesis. Understanding the dynamic regulatory mechanisms of palmitoylation and depalmitoylation may facilitate the development of targeted therapeutic strategies for PD.

## Introduction

Parkinson’s disease (PD) is a progressive neurodegenerative disorder characterized by motor symptoms resulting from α-synuclein (α-Syn) deposition and the loss of dopaminergic neurons (DA), which also contribute to non-motor and cognitive impairments (1). The incidence and prevalence of PD increase considerably with age, thereby greatly contributing to the global public health burden (2). Although substantial progress has been made in elucidating the genetic and molecular physiology underlying PD pathogenesis, a definitive cure has yet to be developed (3). Within the nervous system, protein palmitoylation, a critical post-translational modification (PTM), has gained increasing attention in recent research (4). S-palmitoylation, a lipid modification, serves as a key mechanism facilitating protein translocation to cellular and organelle membranes (5, 6). Qiangqiang He and colleagues proposed that mitochondria-associated membranes (MAMs), which are involved in regulating protein palmitoylation, could serve as potential therapeutic targets for neurodegenerative disorders (7). Among the various proteins involved in palmitoylation regulation, synaptotagmin-11 (Syt11), a member of the 17-protein synaptotagmin family, plays a distinctive role in PD pathogenesis. It has been demonstrated that the palmitoylation levels of synaptic proteins significantly influence the aggregation of pathological proteins in PD (8). Research by Ho and colleagues showed that when Syt11 undergoes palmitoylation within neurons, it becomes selectively anchored to digitonin-insoluble membrane regions. This modification confers resistance to lysosomal degradation and facilitates α-Syn binding to intracellular membranes, thereby promoting its pathological aggregation in PD (8).

Furthermore, approximately 10% of proteins in the human body undergo palmitoylation and are involved in numerous intracellular physiological functions (9). The thioester bond formed by palmitoylation can be cleaved by depalmitoylating enzymes known as acyl-protein thioesterases (APTs), rendering the process reversible and responsive to extracellular signals (10, 11). Overall, palmitoylation is implicated in key cellular functions including signal transduction, differentiation, transcriptional regulation, and metabolism (12). Therefore, a comprehensive understanding of protein palmitoylation may provide new insights into the mechanisms underlying neurodegenerative diseases such as PD.

## Palmitic acid

Palmitic acid (16:0; PAM) is a saturated fatty acid with 16 carbon atoms, comprising approximately 50% of the total saturated fatty acids in the human brain (13). It provides structural support to membrane phospholipids, serves as an energy source, and contributes to protein stabilization (14). Recent evidence suggests that both exogenous dietary intake and endogenous biosynthesis of fatty acids can serve as sources of substrates for protein palmitoylation, thereby influencing protein function (14). Palmitoylation, a reversible PTM, involves the covalent attachment of palmitic acid to cysteine residues on proteins. This process is regulated by palmitoyl transferases (PATs), which catalyze the addition of PAM, and APTs, which mediate its removal (12, 15). Although the brain is rich in PAM, investigations into its metabolic levels remain limited. In mammals, the primary PAT family comprises 23 DHHC (Asp-His-His-Cys motif) proteins, which are essential for palmitoylation activity (16).

Studies have quantified palmitic acid methyl ester (PAM) levels in postmortem human brains, including those of individuals with PD. One study found no significant differences in PAM levels in the occipital and temporal cortices between PD patients and controls (17), However, another study reported a 28% increase in PAM levels in the gray matter of the frontal cortex in sporadic PD cases compared to controls (18). suggesting altered PAM expression in PD-affected regions. In a separate experiment, m-Thy1 transgenic mice (a PD model) fed a PAM-rich diet exhibited increased expression of α-Syn and tyrosine hydroxylase in the brain, accompanied by a decrease in DA levels (19). Hence, increased PAM supply might impact the levels of proteins and neurochemicals, which are crucial for many neurodegenerative diseases (19). These findings imply that elevated PAM intake may influence the expression of key proteins and neurotransmitters involved in neurodegenerative processes. Specifically, increased PAM consumption may upregulate α-Syn and reduce DA levels, both of which are critical factors in the development and progression of PD. The deposition of α-Syn, abnormal PTMs, and DA depletion collectively play pivotal roles in PD pathology.

As a biologically important fatty acid, PAM has physiological functions beyond its established roles in energy metabolism and membrane structure formation. Recent studies have demonstrated that PAM plays a critical role in neurodegenerative diseases by modulating transcription factor activity (20). PAM may exert its influence either through direct modification of transcription factors, such as Nrf2 and CLOCK, or through indirect regulation of inflammatory and oxidative stress pathways, including NF-κB and IRF3, both of which are implicated in neurodegenerative disease mechanisms. On the one hand, excessive intake of PAM has been associated with enhanced inflammation and metabolic dysfunction (21). On the other hand, therapeutic modulation of palmitoylation dynamics, such as inhibiting ZDHHC enzymes or enhancing depalmitoylase activity, has emerged as a potential strategy for disease intervention (22). Future studies should aim to elucidate the transcriptional regulatory networks associated with PAM in specific brain regions and across distinct disease stages. Thus, it is important to investigate whether alterations in PAM expression can dynamically influence protein palmitoylation and thereby contribute to the pathophysiology of PD.

## Correlation between α-synuclein and synaptotagmin-11 with palmitoylation

### Effects of palmitoylation on α-synuclein

In the central nervous system, α-Syn is a small cytosolic protein that is abundantly expressed and closely linked to several neurodegenerative diseases, including PD (23). Research indicates that α-Syn plays a broad regulatory role in signal transduction and contributes to downstream neuroinflammatory processes (24, 25). The absence of α-Syn has been shown to impair PAM uptake by astrocytes, which in turn leads to enhanced secretion of pro-inflammatory cytokines by microglia (26). Moreover, α-Syn deficiency results in elevated cholesterol and cholesterol ester levels in astrocytes and brain tissues (27). Functionally, α-Syn is known for its role as a molecular chaperone during vesicle fusion and neurotransmitter exocytosis. However, the precise toxic mechanisms of α-Syn in PD remain controversial. Membrane binding is central to α-Syn’s physiological role, as it influences the balance between physiological tetramers and aggregation-prone monomers (28, 29). Dysregulation of α-Syn has been implicated in multiple pathogenic pathways, particularly those involving vesicular transport (30). Although α-Syn plays a role in vesicle trafficking, it cannot be directly palmitoylated due to the absence of cysteine residues (31). Therefore, palmitoylation likely influences α-Syn homeostasis indirectly via intermediary proteins. Notably, research indicates that increased palmitoylation of Syt11 in neurons enhances Syt11 abundance and promotes α-Syn membrane binding, contributing to its pathological aggregation (8).

### Palmitoylation of Syt11 at Cys39 and Cys40 enhances its stability

**Figure 1:**
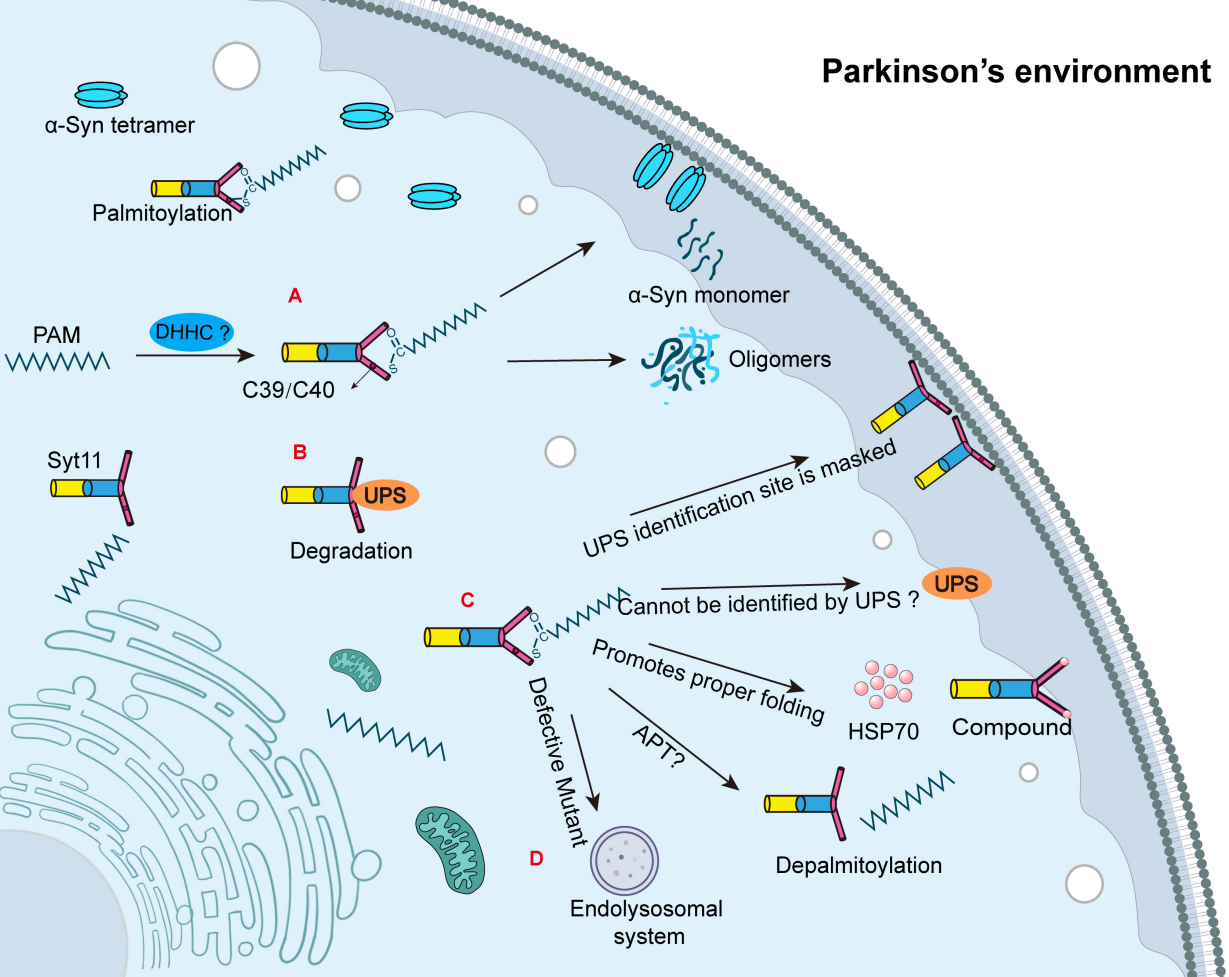
The related mechanisms of synaptic binding protein-11 palmitoylation. **(A)** DHHCs are palmitoyl transferases responsible for palmitoylation. Syt11 undergoes palmitoylation at Cys39/Cys40; however, the specific catalytic DHHC remains unknown. The palmitoylation of Syt11 increases the tetrameric form of α-Syn, which promotes membrane localization and reduces the abundance of α-Syn tetramers. Palmitoylation of Syt11 facilitates the conversion of α-Syn tetramers to the monomeric form of α-Syn, which is more prone to aggregation and ultimately leads to the formation of Lewy bodies and accelerating the progression of Parkinson’s disease (PD). **(B)** Syt11 is degraded by the ubiquitin-proteasome system (UPS). **(C)** Palmitoylation may promote the proper folding of Syt11 by aiding its association with the membrane, facilitating complex formation with HSP70, and potentially resisting UPS degradation. APTs are acyl-protein thioesterases that mediate depalmitoylation, but the specific APT responsible for Syt11 depalmitoylation remains unknown. **(D)** Recent studies have shown that palmitoylation-deficient mutants of Syt11 are degraded by the endolysosomal system.

Synaptotagmins (Syt) are distributed throughout neurons and play essential roles in initiating vesicle fusion with the plasma membrane and mediating neurotransmitter release (32). Syt11, a member of the 17-protein synaptotagmin family, contains a short luminal domain, a transmembrane segment, and two C2 calcium-binding domains (32). Studies have confirmed that Syt11 undergoes palmitoylation in both mouse and human brain tissue, as well as in cultured cortical neurons, and this modification disrupts α-Syn homeostasis in neurons (8). Synaptotagmin-11, encoded by the PD risk gene *SYT11*, has been shown to reduce physiological α-Syn tetramer formation in a palmitoylation-dependent manner (8). Within neurons, palmitoylation of Syt11 increases its protein stability and enhances α-Syn’s binding to cellular membranes, thereby decreasing tetramers and promoting the accumulation of aggregation-prone monomers. Notably, this effect is reproduced by overexpression of wild-type Syt11, whereas palmitoylation-deficient mutants do not exhibit the same influence (8). These findings suggest that palmitoylation-induced upregulation of Syt11 contributes to α-Syn pathology in PD (**Figure 1A**).

Two other synaptotagmins, Syt1 and Syt7, are also subject to post-translational modification at cysteine residues (33). One study reported that Cys117 (corresponding to human Cys188) is a palmitoylation site in Syt11 in the mouse forebrain (34). However, a different result was obtained in a separate investigation. In that study, human wild-type Syt11 tagged with FLAG (Syt11-wt-FLAG) was mutated at Cys39 and Cys40, which were replaced with serine residues and transfected into human embryonic kidney (HEK) cells. Wild-type Syt11 preserved a stable palmitoylation signal (8). Individual mutation of Cys39 or Cys40 weakened the signal but did not abolish it entirely, suggesting that palmitoylation at these sites may occur cooperatively. In contrast, regulation of the Cys188 site had no detectable effect on the palmitoylation signal (8).

While it is established that palmitoylation promotes the stability of Syt11, the underlying mechanisms remain incompletely understood. This review proposes several possible mechanisms by which palmitoylation may enhance Syt11 stability. First, palmitoylation decreases the proportion of cytoplasmic Syt11 and enhances its membrane localization. This shift reduces its exposure to the ubiquitin-proteasome system (UPS), as the cytosolic form is more susceptible to ubiquitination and subsequent degradation (**Figure 1B**). Second, palmitoylation may induce conformational changes in Syt11 that either mask key ubiquitination sites or hinder recognition by E3 ubiquitin ligases (**Figure 1C**). However, this remains speculative and lacks definitive experimental confirmation. Third, palmitoylation appears to enhance the binding of Syt11 to heat shock protein 70 (HSP70), supporting proper protein folding and promoting complex formation within specialized membrane domains, thereby conferring resistance to UPS-mediated degradation (**Figure 1C**).

Experimental findings support the functional importance of these mechanisms. In both rat and human induced pluripotent stem cells, expression of Syt11 was found to be five-fold higher when its cysteine residues (Cys39 and Cys40) remained unmutated, indicating that palmitoylation enhances protein stability (8). In contrast, models expressing serine-substituted Syt11 mutants exhibited reduced protein levels, likely due to accelerated degradation. In rat neurons, the half-life of exogenously expressed Syt11-wt-FLAG protein treated with the protein synthesis inhibitor cycloheximide (CHX) was approximately 16 hours (8). However, the half-life dropped dramatically in the palmitoylation-deficient mutant (CS mutant), with over 80% of the protein degraded within 4 hours (8). These results suggest that palmitoylation is crucial for maintaining Syt11. Although it is known that palmitoylation requires enzymatic activity from palmitoyltransferases, the specific DHHC enzyme responsible for catalyzing Syt11 palmitoylation remains unidentified. Given the review’s subsequent discussion on NLRP3 inflammasome regulation, it is plausible that different DHHC enzymes target specific cysteine sites, mediating distinct biological outcomes. Identifying the regulatory factors and specific DHHC enzymes involved in Syt11 palmitoylation is therefore critical for understanding and precisely modulating its function in PD.

### Syt11 regulates α-synuclein homeostasis through palmitoylation

Palmitoylation not only regulates the biochemical properties of proteins but also alters the biophysical characteristics of membranes. By interacting with membrane phospholipids through its fatty acid chains, palmitoylation can induce local curvature of the lipid bilayer via hydrophobic interactions and steric effects, thereby influencing membrane microenvironments (35, 36). α-Syn preferentially binds to highly curved membranes, such as those found on vesicle surfaces, via its amphipathic helical structure. This interaction is integral to its role in vesicle trafficking and membrane dynamics (37–39). Considering the known influence of Syt11 on α-Syn function, several studies have investigated whether a direct interaction exists between the two proteins. Current evidence suggests that Syt11 may not physically interact with α-Syn. Instead, Syt11 appears to regulate the membrane-binding capacity and homeostasis of α-Syn indirectly through a palmitoylation-dependent mechanism. This regulation likely involves changes to local membrane properties, such as lipid composition or curvature, rather than direct protein-protein interaction (8).

Importantly, Syt11 is also known to play a role in vesicle transport, and its palmitoylation is emerging as a potential molecular link to α-Syn function (40). Through multidimensional molecular mechanisms—including membrane anchoring, oligomerization regulation, and vesicle transport remodeling—palmitoylation is a key regulator of α-Syn-mediated cytopathology in PD (41). Additionally, palmitoylation regulates the membrane targeting of essential vesicle-associated proteins such as SNAP25 (22), which function synergistically with α-Syn to ensure proper synaptic vesicle fusion and dynamics.

### Syt11 degradation and depalmitoylation

The dynamic attachment and removal of palmitic acid from proteins are mediated by palmitoyl acyltransferases (PATs) and APTs, respectively (42). This reversibility makes palmitoylation a compelling target for drug development and gene therapy. Recent studies show that palmitoylation-deficient mutants of Syt11 undergo rapid degradation via the endolysosomal pathway, and proteasome inhibition does not rescue protein levels, suggesting the proteasome is not involved in this context (**Figure 1D**) (8). This finding contrasts with earlier work indicating that Syt11 is ubiquitinated by parkin and degraded via the proteasome pathway (43). In other neurological diseases, preclinical and clinical studies suggest that mimicking the activity of depalmitoylating enzymes—such as palmitoyl-protein thioesterase 1 (PPT1)—may offer therapeutic benefit. Treatments using the depalmitoylating agent *N*-tert-butylhydroxylamine (NtBuHA) or gene therapy approaches to replace defective *PPT1* variants have demonstrated success in disease models (44). For instance, a six-month PPT1 mimetic treatment significantly reduced microglial activation in *Ppt1* ^−^/^−^ mice, a model of Infantile Neuronal Ceroid Lipofuscinosis (45). Several inhibitors have been developed to target depalmitoylation: palmostatin B inhibits both APT1 and APT2, while ML348 and ML349 selectively inhibit APT1 and APT2, respectively (46, 47). However, blocking APT1 and APT2 does not impair palmitate removal from postsynaptic density protein PSD-95. Instead, the hydrolase domain-containing protein ABHD17 serves as the specific depalmitoylase for PSD-95 (48). New members of the APT family, such as ABHD10 and ABHD17, belong to the α/β hydrolase superfamily, suggesting a broader depalmitoylase landscape beyond the canonical APT1 and APT2 (48). Depalmitoylases display organelle-specific localization. APT1 and ABHD10 function within mitochondria (49, 50), while ABHD17 isoforms (A/B/C) localize to the plasma membrane and endosomal compartments (48). Future research should aim to identify the specific APT(s) responsible for regulating Syt11 depalmitoylation and to elucidate their mechanistic roles. This may offer novel insights for targeted PD therapy.

## Palmitoylation promotes NLRP3 inflammasome assembly

### The activation of the NLRP3 inflammasome triggers inflammation in microglial cells

The NLRP3 inflammasome plays a central role in microglial-mediated inflammation in PD. As early as 1988, McGeer and colleagues identified HLA-DR^+^ reactive microglia in postmortem brain tissue of PD patients, establishing a foundational link between inflammation and PD pathogenesis (51). The core pathological features of PD include abnormal aggregation of α-Syn and the loss of DA (52). Increasing evidence suggests that excessive microglial activation and the pathological release of pro-inflammatory mediators contribute to neuronal degeneration (53). Remarkably, microglial activation begins early in PD, preceding both α-Syn aggregation and Lewy body formation, and may persist throughout disease progression (54, 55). Consequently, therapeutic strategies aimed at modulating inflammation in PD have garnered significant interest. One proposed mechanism for microglial activation involves lysosomal dysfunction caused by PD-related gene mutations, which impairs glial phagocytic capacity and amplifies inflammatory responses (56).

**Figure 2:**
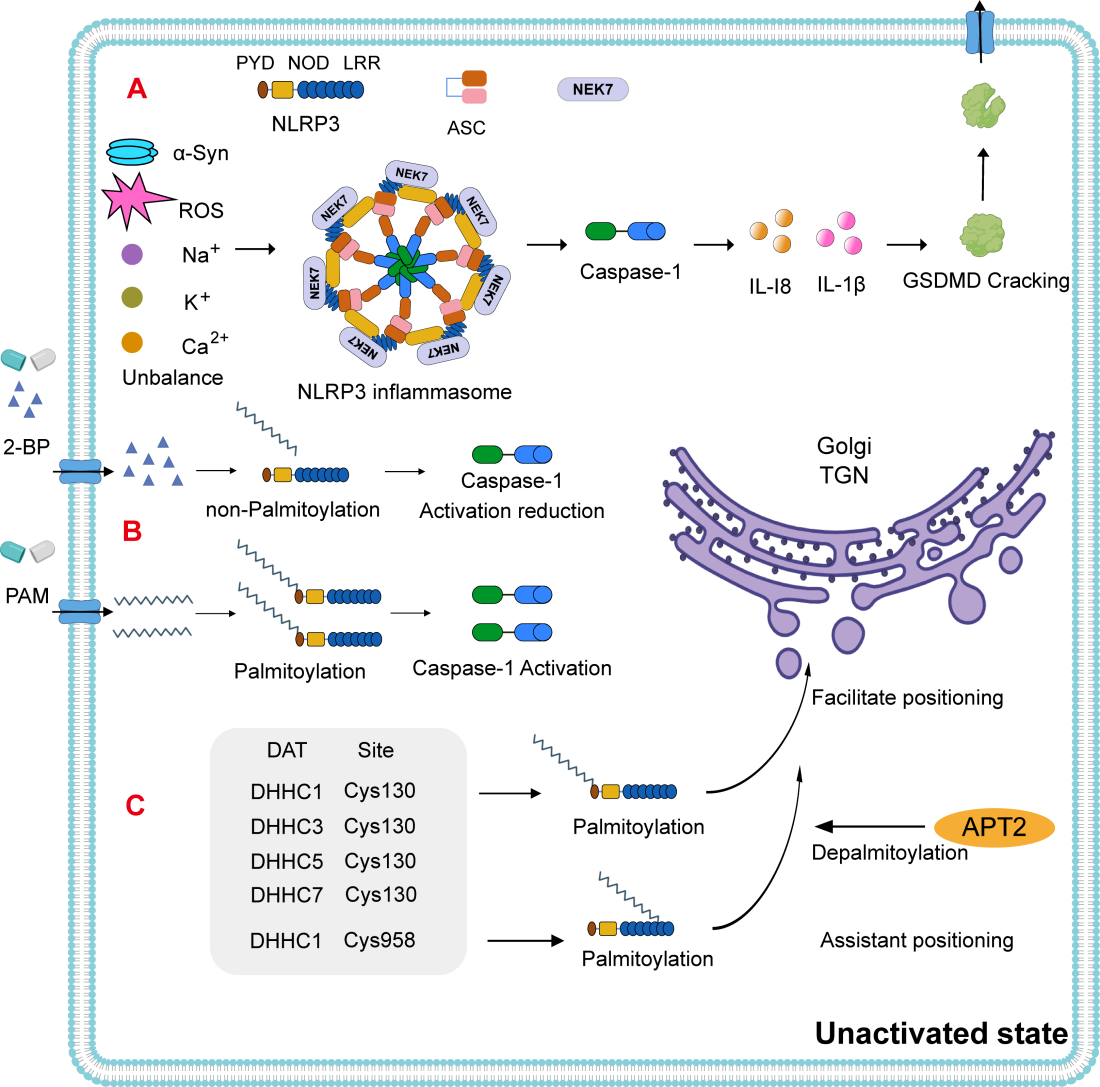
NLRP3 inflammasome activation pathway. **(A)** NLRP3 consists of an N-terminal PYD domain, a central ATPase domain (known as the nucleotide-binding oligomerization domain, NOD), and a C-terminal leucine-rich repeat (LRR) domain. In PD, the NLRP3 inflammasome is activated via intracellular ion homeostasis imbalances such as α-Syn, reactive oxygen species (ROS), sodium (Na^+^), calcium (Ca^2+^), and potassium (K^+^). The assembly of the NLRP3 inflammasome leads to the activation of caspase-1. This, in turn, induces the maturation and release of IL-1 and IL-18, as well as the cleavage of gasdermin D (GSDMD), promoting cell death. **(B)** Notably, using 2-bromopalmitate (2-BP) to block palmitoylation results in reduced caspase-1 activation, a phenomenon that can be reversed by the addition of exogenous palmitate. **(C)** In the resting state of NLRP3, DHHC1/3/5/7 can facilitate its transfer to the trans-Golgi network (TGN) at Cys130, with the thioesterase APT2 inhibiting this process (70–72). Typically, resting-state NLRP3 is insufficient to recruit itself to the TGN. DHHC1 adds a PAM group at the Cys958 site to assist in the additional recruitment of NLRP3 to the TGN.

Moreover, structural alterations and mutations in extracellular α-Syn can directly trigger microglial activation (57). As innate immune cells of the central nervous system, microglia are particularly efficient at internalizing and degrading α-Syn. The NLRP3 inflammasome has been implicated in α-Syn-induced inflammation, acting as a molecular hub for the amplification of neuroinflammatory signaling (58, 59). In microglia, the NLRP3 inflammasome is a multiprotein complex composed of a sensor (NLRP3), an adaptor (ASC), and an effector (caspase-1). Structurally, NLRP3 comprises an N-terminal pyrin domain (PYD), a central nucleotide-binding oligomerization (NOD) domain, and a C-terminal leucine-rich repeat (LRR) domain (60). Upon assembly, the inflammasome activates caspase-1, which cleaves gasdermin D (GSDMD) and promotes the maturation and release of pro-inflammatory cytokines such as interleukin-1β (IL-1β) and IL-18 (**Figure 2A**) (61). This cascade culminates in pyroptosis, a form of lytic, inflammation-driven cell death.

### Palmitoylation as a novel activation pathway for the NLRP3 inflammasome

The release of NLRP3 inflammasomes and downstream inflammatory cytokines has been detected in both the substantia nigra pars compacta (SNpc) and the peripheral plasma of PD patients (58). This highlights the important role of the NLRP3 inflammasome in PD pathogenesis and underscores the need to investigate its activation mechanisms in microglia. NLRP3 activation occurs via canonical and non-canonical pathways (59), and in PD, α-Syn is known to initiate the activation of microglial NLRP3 inflammasomes. Disruptions in intracellular ionic homeostasis—including reactive oxygen species (ROS), sodium (Na^+^), calcium (Ca^2+^), and potassium (K^+^)—as well as mitochondrial and lysosomal dysfunction, also contribute to NLRP3 activation (62–65) (**Figure 2A**).

Despite advances in understanding NLRP3 activation, existing pathways have not yet succeeded in fully regulating microglial inflammatory states. Emerging research indicates that, in its inactive form, NLRP3 can oligomerize into a double-cage structure and is localized to the trans-Golgi network (TGN) in a palmitoylation-dependent manner (66). Furthermore, fine-tuning of NLRP3 inflammasome activation via palmitoylation has been shown to modulate pyroptotic signaling cascades (67). *In vitro* experiments using 2-bromo-palmitate (2-BP), a competitive palmitate analog that inhibits palmitoylation, demonstrated that caspase-1 activation is negatively correlated to 2-BP concentration in already activated NLRP3-expressing cells. Interestingly, the addition of exogenous palmitate restored caspase-1 activity (68) (**Figure 2B**). These findings suggest that while 2-BP inhibits NLRP3 inflammasome activation, palmitate supplementation can reverse this effect and promote inflammasome-mediated caspase-1 expression. These results reveal a regulatory mechanism that may also be applicable within the nervous system.

### Temporal and spatial mechanisms of NLRP3 palmitoylation

**Figure 3:**
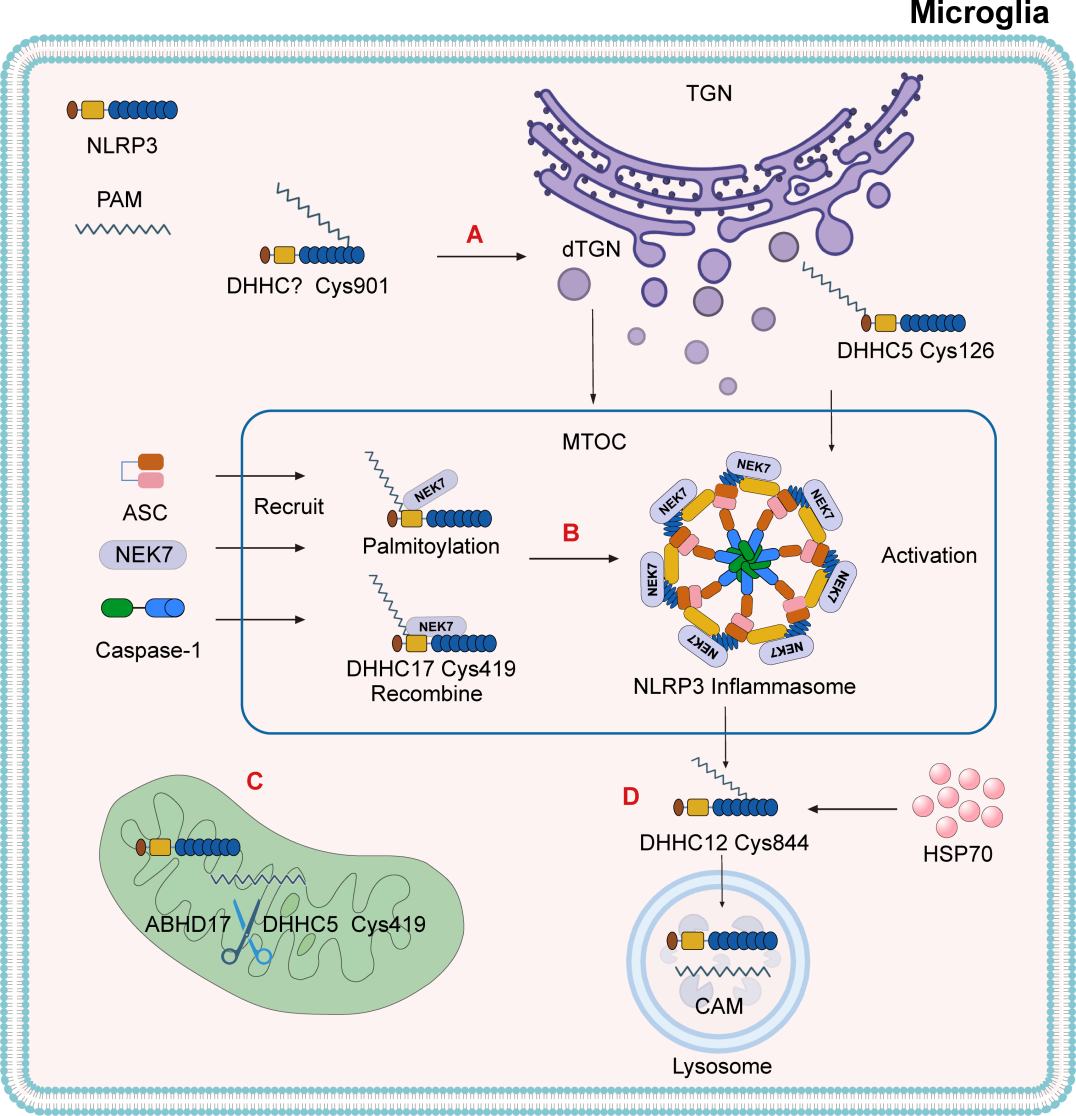
The mechanism by which palmitoylation activates the NLRP3 inflammasome. **(A)** Under the action of PAT, NLRP3 undergoes palmitoylation at the Cys901 site, which leads to the dispersion of the TGN. NLRP3 is recruited to the dispersed TGN (dTGN) via ionic bonds between its polybasic region and phosphatidylinositol-4-phosphate (PtdIns4P). The dTGN acts as a scaffold for NLRP3, transporting it to the microtubule-organizing center (MTOC). The palmitoylation of NLRP3 at the cysteine Cys126 site, catalyzed by DHHC5, is also a modification required for inflammasome activation. **(B)** DHHC17 triggers the palmitoylation of NLRP3 at Cys419, promoting the formation of the NLRP3-NEK7 complex and recruiting ASC and Caspase-1 at the MTOC to form the NLRP3 inflammasome. After the NLRP3-NEK7 complex is formed, DHHC17 mediates palmitoylation at Cys419, allowing NLRP3 to be re-associated with the opposite site of NEK7. **(C)** The thioesterase ABHD17 can reverse the catalytic effect of DHHC5 on NLRP3. **(D)** After inflammasome activation, DHHC12 triggers the palmitoylation of the NLRP3 Cys844 residue, promoting its binding to HSC70. This transports the substrate to lysosomes and facilitates the degradation of NLRP3 through the chaperone-mediated autophagy (CMA) pathway.

In its resting state, NLRP3 is translocated to the TGN via palmitoylation at the Cys130 residue, a process catalyzed by DHHC1, DHHC3, DHHC5, and DHHC7 (**Figure 2C**). The thioesterase APT2 inhibits this localization by removing palmitoyl groups (69–71). The NLRP3 polypeptide alone lacks the ability to recruit the protein to the TGN; however, DHHC1-mediated palmitoylation at Cys958 enhances NLRP3’s recruitment to this compartment (71). Upon activation, palmitoylation occurs at the Cys901 site via PAT activity, prompting TGN disassembly (**Figure 3A**). Activated NLRP3 is then recruited to the dispersed TGN (dTGN) via ionic interactions between its polypeptide region and phosphatidylinositol-4-phosphate (PtdIns4P) (72). The dTGN serves as a platform for NLRP3 aggregation and facilitates its transport to the microtubule-organizing center (MTOC) (73), where NLRP3 assembles with NEK7 and ASC to form the active inflammasome complex (74) (**Figure 3B**). Following recruitment, palmitoylation supports inflammasome assembly and stabilizes NLRP3-NEK7 interactions, enhancing caspase-1 activation and the subsequent release of mature IL-1β and IL-18. Notably, the thioesterase ABHD17 can counteract the DHHC5-mediated palmitoylation of NLRP3, highlighting a reversible regulatory step in inflammasome assembly (75) (**Figure 3C**). Furthermore, DHHC17 mediates palmitoylation at the Cys419 site of NLRP3, allowing re-binding via the NACHT domain and contributing to inflammasome stability (68).

After inflammasome activation, additional palmitoylation at Cys844—catalyzed by DHHC12—promotes NLRP3’s interaction with HSC70, targeting the complex for lysosomal degradation via chaperone-mediated autophagy (CMA) (**Figure 3D**) (76). Recent studies have also demonstrated that palmitoylation at Cys126 by DHHC5 is critical for proper subcellular localization and activation of the NLRP3 inflammasome (77). These findings collectively indicate that palmitoylation is essential for both the assembly and degradation of the NLRP3 inflammasome and that α-Syn-mediated microglial activation promotes DA neuron degeneration via this pathway. Current knowledge of DHHC protein localization is primarily derived from co-expression analyses. At least 23 mammalian DHHC enzymes display distinct subcellular localization patterns (78, 79). These spatial and temporal differences may serve as auxiliary evidence supporting the regulatory role of palmitoylation in NLRP3 inflammasome dynamics.

## Potentially relevant proteins undergoing palmitoylation

### DA neurons

Parkinson’s disease is characterized by the progressive loss of DA neurons in the SNpc, which project to the striatum. This neurodegenerative loss results in a deficiency of DA in the nigrostriatal pathway, a critical component of motor control, thereby leading to the hallmark motor symptoms of PD (80). Current treatment strategies primarily aim to alleviate motor symptoms through DA replacement therapy, including carbidopa/levodopa and dopamine agonists (81). However, prolonged pharmacological intervention can result in debilitating motor complications in a subset of patients (82). Emerging cellular therapies, such as the use of induced pluripotent stem cells, show considerable promise in PD treatment, although patient responses remain variable (83). Advancing our understanding of DA regulation and signaling could uncover novel therapeutic strategies to enhance DA production or release in surviving SNpc neurons.

Research has demonstrated that the dopamine transporter (DAT) undergoes palmitoylation, and defects in this modification reduce both its stability and function (84). Among 12 tested PATs, DHHC2, DHHC3, DHHC8, DHHC15, and DHHC17 were found to promote DAT palmitoylation, with the modification localized to Cys580 (85). In human DAT (hDAT), palmitoylation at Cys581 facilitates the formation of stable, energetically favorable dimers that influence dopamine uptake efficiency (86). Functionally, DAT palmitoylation increases the maximum rate of dopamine uptake (Vmax) and decreases its degradation, while depalmitoylation enhances dopamine efflux, reduces Vmax, and promotes lysosome-mediated degradation. DAT is depalmitoylated by APT1 and APT2, members of the serine hydrolase superfamily, and degraded by lysosomal pathways involving PPT1 (85, 87). In the striatum, ZDHHC15 has been identified as a mediator of DAT palmitoylation (88). Deficiencies in this process compromise DAT function and reduce DA reuptake at synapses, although this impairment is typically transient and reversible (88, 89). Further investigation is required to clarify the regulatory mechanisms involving ZDHHC15 and to determine whether targeting this enzyme may offer a therapeutic strategy to enhance dopamine transport in PD.

### DJ-1

Although palmitoylation of proteins encoded by *PARK* genes and PD-associated risk loci has not been extensively studied, available evidence suggests that certain PARK proteins undergo palmitoylation. One such protein is DJ-1, encoded by the *PARK7* gene, which has been shown to be palmitoylated (90). Under basal conditions, DJ-1 is predominantly localized in the cytoplasm but is also present in mitochondria and the nucleus (91, 92). DJ-1 contains three cysteine residues at positions 46, 53, and 106 in the N-terminal region (93).

DJ-1 is associated with membrane lipid rafts (LRs) in both astrocytes and neurons, a process that depends on the palmitoylation of its cysteine residues. Furthermore, palmitoyl-proteomic studies conducted in human cell lines have identified palmitoylation in additional PD-related proteins, including ubiquitin C-terminal hydrolase L1 (UCHL1), encoded by *PARK5*, and lysosomal acid β-glucosidase (GBA1), a known PD risk factor (94). However, the palmitoylation status of UCHL1 and GBA1 has not yet been validated through follow-up experiments.

## Discussion and outlook

α-Synuclein is a small, highly expressed cytosolic protein whose pathological aggregation contributes to the progression of PD. Its membrane fusion activity and affinity for vesicular membranes are key to its physiological function, as they influence the dynamic equilibrium between physiological α-Syn tetramers and aggregation-prone monomers (8). While α-Syn lacks cysteine residues and therefore cannot be directly palmitoylated, recent findings suggest that its membrane-binding stability is modulated by the palmitoylation of synaptotagmin-11 (Syt11), a synaptic binding partner. Palmitoylated Syt11 has been shown to reduce α-Syn tetramers, a change potentially linked to PD pathogenesis (8). The palmitoylation of Syt11 requires intact cysteine residues at positions Cys39 and Cys40. Although these sites are critical for maintaining α-Syn homeostasis, their individual functional contributions remain indistinguishable (8). While it is established that palmitoylation enhances the stability of Syt11, this post-translational modification depends on catalysis by a palmitoyl acyltransferase (DHHC). To date, the specific DHHC enzyme responsible for Syt11 palmitoylation has not been identified.

From a therapeutic standpoint, modulating the palmitoylation cycle of Syt11 may provide a novel approach for PD treatment. Inhibiting or silencing the DHHC enzyme involved in Syt11 palmitoylation could potentially restore physiological α-Syn tetramer formation. One promising direction is the targeting of GNS561, a selective inhibitor of PPT1, currently under investigation for cancer therapy (95). Although GNS561 has demonstrated the ability to cross the blood-brain barrier (BBB) (95), its impact on the cerebral palmitoyl-proteome remains to be validated. The development of highly selective agents capable of modulating protein-specific palmitoylation and depalmitoylation within the brain is a critical step toward advancing therapies for neurodegenerative disorders.

In parallel, the palmitoylation of NLRP3 has been shown to regulate its localization to the TGN. The use of 2-BP, a palmitate analog, competitively inhibits palmitoylation and thereby modulates inflammasome activity (68). Notably, caspase-1 activation decreases with reduced 2-BP concentration, while the subsequent addition of palmitate restores its expression. This finding underscores the role of palmitoylation in controlling NLRP3 inflammasome activation. The resting state of NLRP3 is regulated by palmitoylation at Cys130, catalyzed by DHHC1, DHHC3, DHHC5, and DHHC7. Simultaneously, DHHC1 facilitates recruitment of NLRP3 to the TGN through palmitoylation at Cys958 (71). Upon activation, NLRP3 is palmitoylated at Cys901, triggering the fragmentation of the TGN into the dTGN. This structural transformation enables NLRP3 to interact with NEK7 and ASC at the MTOC, forming the inflammasome complex (74). These observations reinforce the idea that palmitoylation is not a uniform process but rather one that exhibits protein- and context-specific effects.

Despite these advances, several critical questions remain. For instance, although palmitoylation at NLRP3 Cys901 has been shown to facilitate its translocation to the dTGN and promote inflammasome activation (72), the PAT responsible for this site-specific modification has yet to be identified. Understanding how each of the 23 mammalian DHHC family members selectively recognize and modify distinct cysteine sites on NLRP3 could refine therapeutic targeting strategies. Additionally, the spatial and temporal sequence of palmitoylation events on NLRP3, and their respective biological consequences, deserve further detailed investigation.

Selective inhibition of PSD-95 depalmitoylation has been proposed as a viable therapeutic strategy in the context of Alzheimer’s disease (AD) (96). Although PSD-95’s specific role in PD remains unexplored, its critical involvement in synaptic function suggests that it may also serve as a potential therapeutic target for PD. In addition to postsynaptic proteins, dopamine transporters (DATs) also contain modifiable cysteine residues. Palmitoylation of DAT enhances the maximum velocity (Vmax) of dopamine uptake, while depalmitoylation increases dopamine efflux and reduces Vmax. Although the precise mechanisms remain unclear, these functional changes underscore the regulatory potential of palmitoylation in DA neurotransmission.

Moreover, several PD-associated risk genes encode proteins that are either directly or indirectly influenced by palmitoylation. In summary, palmitoylation is increasingly recognized as a key regulatory mechanism in PD pathogenesis. A comprehensive understanding of its molecular functions and dynamics could facilitate the development of novel interventions by targeting the palmitoylation–depalmitoylation cycle, thereby offering new therapeutic strategies for PD.
